# Regeneration of Spinal Cord Connectivity Through Stem Cell Transplantation and Biomaterial Scaffolds

**DOI:** 10.3389/fncel.2019.00248

**Published:** 2019-06-06

**Authors:** Hiroyuki Katoh, Kazuya Yokota, Michael G. Fehlings

**Affiliations:** ^1^Division of Genetics and Development, Krembil Research Institute, Toronto, ON, Canada; ^2^Department of Orthopaedic Surgery – Surgical Sciences, School of Medicine, Tokai University, Tokyo, Japan; ^3^Department of Orthopaedic Surgery, Graduate School of Medical Sciences, Kyushu University, Fukuoka, Japan; ^4^Institute of Medical Science, University of Toronto, Toronto, ON, Canada; ^5^Division of Neurosurgery, University of Toronto, Toronto, ON, Canada; ^6^Spine Program, Toronto Western Hospital, University Health Network, Toronto, ON, Canada

**Keywords:** traumatic spinal cord injury, central nervous system, regeneration, stem cell transplantation, biomaterials

## Abstract

Significant progress has been made in the treatment of spinal cord injury (SCI). Advances in post-trauma management and intensive rehabilitation have significantly improved the prognosis of SCI and converted what was once an “ailment not to be treated” into a survivable injury, but the cold hard fact is that we still do not have a validated method to improve the paralysis of SCI. The irreversible functional impairment of the injured spinal cord is caused by the disruption of neuronal transduction across the injury lesion, which is brought about by demyelination, axonal degeneration, and loss of synapses. Furthermore, refractory substrates generated in the injured spinal cord inhibit spontaneous recovery. The discovery of the regenerative capability of central nervous system neurons in the proper environment and the verification of neural stem cells in the spinal cord once incited hope that a cure for SCI was on the horizon. That hope was gradually replaced with mounting frustration when neuroprotective drugs, cell transplantation, and strategies to enhance remyelination, axonal regeneration, and neuronal plasticity demonstrated significant improvement in animal models of SCI but did not translate into a cure in human patients. However, recent advances in SCI research have greatly increased our understanding of the fundamental processes underlying SCI and fostered increasing optimism that these multiple treatment strategies are finally coming together to bring about a new era in which we will be able to propose encouraging therapies that will lead to appreciable improvements in SCI patients. In this review, we outline the pathophysiology of SCI that makes the spinal cord refractory to regeneration and discuss the research that has been done with cell replacement and biomaterial implantation strategies, both by itself and as a combined treatment. We will focus on the capacity of these strategies to facilitate the regeneration of neural connectivity necessary to achieve meaningful functional recovery after SCI.

## Introduction

Spinal cord injury (SCI) is a severely debilitating condition leading to neurological dysfunction, loss of independence, respiratory failure, psychological morbidities, and an increased lifelong mortality rate (Marion et al., 2017; Satkunendrarajah et al., 2018; Shibahashi et al., 2018; Wang et al., 2018b). In the United States, approximately 288,000 individuals are estimated to suffer from symptoms caused by SCI, and a recent survey showed the annual incidence of SCI is approximately 54 cases per one million people (Fehlings et al., 2018). Worldwide, the estimated incidence of SCI ranges from 250,000–500,000 individuals per year (Singh et al., 2014). The main causes of SCI are motor vehicle accidents, falls, and violent acts, but with the aging of the population in many industrialized countries, the SCI patient profile is slowly evolving toward more elderly SCI patients injured through falls (Sekhon and Fehlings, 2001). SCI has a tremendous impact on the personal, professional, and social life of patients, imposing enormous psychological and financial burdens on the patients and their caregivers (Munce et al., 2016; Backx et al., 2018). The overall lifetime economic costs with complete SCI can exceed $3 million per person, and the estimated economic burden associated with SCI in Canada is approximately $2.67 billion annually (Krueger et al., 2013). This recognition of the personal and social costs of SCI has fostered extensive basic research into the pathology of the injured spinal cord and treatment strategies for SCI. Despite decades of research and numerous regenerative approaches that demonstrated promising results in animal models, the global scientific community has yet to provide SCI patients with a viable option to prevent the devastating outcome of traumatic SCI or to reverse the neurological impairment brought about by the condition. While SCI patients may be frustrated by the lack of an apparent “cure,” there is a palpable anticipation within the circle of SCI researchers that we will soon begin to observe significant functional improvements from clinical trials in the very near future.

Animal studies up until a decade ago had often demonstrated a significant functional improvement with various interventions, citing significantly lower inflammation, smaller cavity size, higher axonal growth, or increased myelination as possible explanations for the observed recovery, but the true reason for the improvement was often left within a black box (Badhiwala et al., 2018). With the accumulating basic knowledge on the fundamental pathophysiology underlying SCI, along with the improvements in techniques and technology to perform increasingly precise analyses on the changes brought about by treatment strategies, we are finally shedding light into this black box. So while the ”cure” may still be out of reach, our enhanced understanding of the obstacles and the hurdles in the path to regenerating connectivity of the neural circuits will hopefully greatly improve the accuracy of our endeavors to improve the function and quality of life of patients with SCI. In this review, we outline the pathophysiology of SCI that makes the spinal cord refractory to regeneration and spontaneous recovery following injury and discuss strategies being explored to reestablish connectivity within the injured spinal cord, focusing on stem cell-based therapy and biomaterial engineering.

## Pathophysiology of SCI

Traumatic injury to the spinal cord can be caused by compressions, lacerations, and contusions, which lead to a spectrum of neurological symptoms depending on the level and the severity of the injury such as motor/sensory dysfunction, autonomic deficits, neuropathic pain, autonomic dysreflexia, and bowel/bladder dysfunction (Furlan et al., 2016; Moonen et al., 2016; Stroman et al., 2016). The processes occurring within the injured spinal cord can be divided according to the elapsed time from the precipitating injury into acute (<48 h), subacute (48 h to 14 days), intermediate (14 days to 3 months), and chronic phases (>3 months) (Aimone et al., 2004; Shechter et al., 2009; Chamankhah et al., 2013; Moghaddam et al., 2015). In order to understand the pathophysiology, cellular composition, inflammatory reaction, and expression of trophic and other factors within the spinal cord after traumatic SCI, it is also helpful to divide the process into primary and secondary injuries.

**FIGURE 1:**
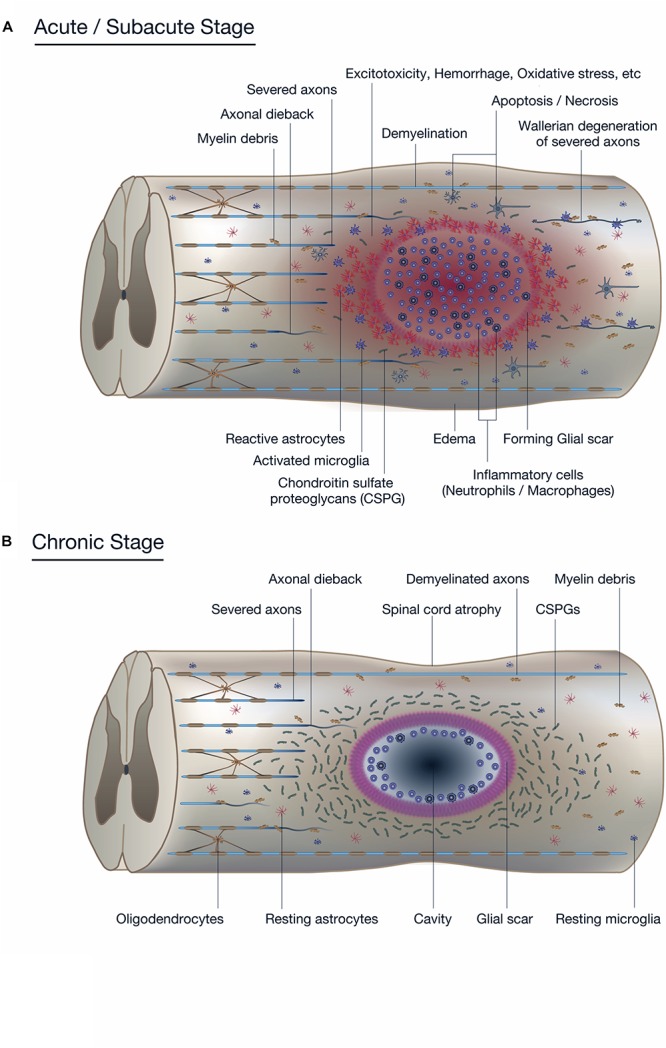
Pathophysiology of spinal cord injury (SCI). **(A)** The diagram shows the pathophysiological events occurring around the lesion site during the acute to subacute phase of SCI. The primary and secondary injury mechanisms lead to inflammation, hemorrhage, apoptosis, and necrosis. Resident neurons, oligodendrocytes, and astrocytes near the lesion are forced into apoptosis or necrosis, resulting in anterograde (Wallerian degeneration) and retrograde (axonal dieback) axonal degeneration. Reactive astrocytes and other glial cells secrete chondroitin sulfate proteoglycans (CSPGs), which acts as a physical and chemical barrier that impedes endogenous tissue repair processes such as axonal sprouting and synaptic reorganization. **(B)** The diagram shows the pathophysiological events in the chronic phase of SCI. In the epicenter of the lesion, a cavitation has occurred that is surrounded by connective scar tissues and contains cerebrospinal fluid (CSF). The phenotype of reactive astrocytes has changed into scar-forming astrocytes that impede regenerating axons from crossing the lesion. Some inflammatory immune cells remain around the lesion even in the chronic phase of SCI.

The initial traumatic event, which may or may not accompany fractures and/or a dislocation of the vertebral column, results in the primary injury through mechanical compression, contusion, stretching, or kinking of the spinal cord (Sekhon and Fehlings, 2001). Neurons, oligodendrocytes, and other components essential for neuronal transmission are physically insulted (Wilcox et al., 2017), and the disrupted vascular components, including the blood-spinal cord barrier (BSCB), induce infiltration of inflammatory cells (Kunis et al., 2013; Shechter et al., 2013; Li et al., 2017). The initial injury triggers a subsequent secondary injury cascade which leads to further chemical and physical damage to the spinal cord and resultant neurological deficits. Increased glutamate results in neuronal excitotoxicity due to the accumulation of intracellular Ca^2+^, leading to an increase in reactive oxygen species (ROS) (Ouardouz et al., 2009; Yin et al., 2012; Breckwoldt et al., 2014) that damage cellular components such as nucleic acids, proteins, and phospholipids, and cause cellular loss and subsequent neurological dysfunction (Khayrullina et al., 2015; von Leden et al., 2017; Figure 1A).

Secondary injury refers to the multifaceted pathological process that begins after primary injury and can last for several weeks, in which increased permeabilization of cells, pro-apoptotic signaling, ischemia, and breakdown of the BSCB further exacerbates insult to the injured spinal cord (Casha et al., 2005; Yu et al., 2009; Yu and Fehlings, 2011; Robins-Steele et al., 2012; Wu et al., 2014). Disrupted blood vessels cause severe hemorrhage (Saiwai et al., 2010; Yokota et al., 2016) and allow infiltration of inflammatory cells including neutrophils, monocytes/macrophages, T cells, and B cells into the spinal cord tissue (Ankeny et al., 2009; Beck et al., 2010; Saiwai et al., 2013; Raposo et al., 2014) that release inflammatory cytokines such as tumor necrosis factor (TNF)-α, interleukin (IL)-1α, IL-1β, and IL-6 (Kumamaru et al., 2012; Nguyen et al., 2012). These cytokines, often reaching their peak 6–12 h after injury, further induce an overwhelming inflammatory response during the acute to subacute phase that expands the lesion in a rostral and caudal direction (Min et al., 2012). Activated microglia and infiltrated macrophages have been shown to be responsible for the necrosis and apoptosis of neurons, astrocytes, and oligodendrocytes residing in the vicinity of the lesion (Chu et al., 2007), further deteriorating the neurological outcome (Figure 1B; Horn et al., 2008; Floriddia et al., 2012). Early measures to decrease inflammation and prevent apoptosis have long been a target intervention for SCI, but the increasing knowledge of the beneficial aspects of the inflammation process following SCI has made it necessary to carefully monitor the effects of inflammation-modulating strategies (Rust and Kaiser, 2017).

## Cell Death in the Injured Spinal Cord

At the lesion site of the injured spinal cord, the death of the constituent cells that make up the neural circuitry, along with the loss of cells tasked with its maintenance, is a fundamental cause of functional impairment. Traditionally, the mechanism of cell death after SCI was characterized as an initial wave of necrosis at the lesion epicenter followed by a delayed phase of cell death in neighboring tissue through necrotic and apoptotic mechanisms (Baptiste and Fehlings, 2006). Necrosis is a passive non-programmed cell death triggered by SCI trauma that causes lethal disruption of cell structure and activity. It involves failure of membrane integrity, mitochondrial damage, rapid loss of ATP, sudden loss of ionic homeostasis, and induction of ROS that leads to organelle as well as cell swelling and terminates with the disposal of cell corpses in the absence of obvious phagocytic and lysosomal involvement. Apoptosis, on the other hand, is an active programmed cell death sequence in which neurochemical changes occur in an orderly fashion and is often dependent on caspase activation. It is characterized by the activation of cell signals directly involved in mitochondrial function, and leads to cytoplasmic shrinkage, chromatin condensation, and nuclear fragmentation, culminating with the formation of apoptotic bodies that are phagocytosed by neighboring cells and degraded within lysosomes (Galluzzi et al., 2018). A key player in apoptosis is caspase-3, a member of the caspase family of cysteine proteases that regulate programmed cell death, which cleaves essential downstream substrates involved in apoptosis. The initiation of the apoptotic pathway following SCI can be mediated by death receptors FAS (CD95) and p75, which activate caspases and initiate the apoptotic pathway in oligodendrocytes, astrocytes, and microglia (Casha et al., 2001, 2005). The caspase-3 apoptotic pathway triggers apoptosis in neurons in the early phase of injury and in oligodendrocytes adjacent to and distant from the lesion hours to days later. Combined with the limited proliferative potential of OPCs, the susceptibility of oligodendrocytes to apoptosis even when they are distant from the lesion leads to a wide area of demyelination, which greatly impairs the function of preserved axons.

The scientific field regarding cell death is evolving, with novel mechanisms that orchestrate multiple cell-death pathways continually being unveiled. The differentiation of the various processes can be difficult, and the Nomenclature Committee on Cell Death has recently published an updated classification of cell death subroutines focusing on mechanistic and essential aspects of the processes (Galluzzi et al., 2018). Multiple processes of cell death have also been reported in SCI, and the traditional understanding of cell death in the injured spinal cord as either necrosis or apoptosis is no longer accurate. One of the additional major players implicated in mediating cell death in SCI is autophagy, which under normal conditions plays an important role in the maintenance of homeostasis by recycling toxic agents, unnecessary proteins, and damaged organelles through an autophagosomal and lysosomal process. When this processing of components through the autophagy system, or autophagy flux, is blocked or overrun by components awaiting processing, the accumulation of dysfunctional autophagosomes damages cells and triggers death (Lipinski et al., 2015). The autophagy pathway is closely linked to endoplasmic reticulum stress, which also plays a role in maintaining cellular homeostasis and triggers apoptosis if endoplasmic reticulum stress exceeds the capacity of its processing mechanism (Kuroiwa et al., 2014). There are still other pathways of cell death that lay outside of the traditionally acknowledged cell death processes in SCI: a type of programmed cell death termed necroptis (Liu et al., 2015), a regulated cell death called parthanatos that is driven by the hyperactivation of the DNA damage response machinery (Kuzhandaivel et al., 2010), and numerous caspase-independent cell death pathways often involving the apoptosis inducing factor (AIF) (Wu et al., 2007).

Many of the pathways involved in cell death after SCI have been studied as possible targets of therapeutic intervention, but the results have been mixed. The inhibition of caspase-3 was examined exhaustively considering its significance in the apoptotic pathway, with some studies showing improvement (Kaptanoglu et al., 2005), while some studies reported no apparent improvement (Ozawa et al., 2002). In fact, the processes that induce cell death seem to be interconnected and complementary, with the minor pathway becoming dominant when the primary pathway is inhibited (Proskuryakov et al., 2003). Therefore, although the cell-death pathway remains an attractive target to reduce loss of neural cells in SCI, it may be more productive to intervene in the processes that trigger cell death rather than the cell death pathway itself.

## Wallerian Degeneration and Axonal Dieback

After SCI, axons and dendrites that lose connection to their original neural pathways degenerate from the site of injury in a direction away from the injury epicenter: (Bareyre et al., 2005; Kerschensteiner et al., 2005) the anterograde degenerative process is called Wallerian degeneration, while the retrograde degeneration of axons is referred to as axonal dieback. The spinal cord mass decreases both rostral and caudal to the lesion following SCI, suggesting that the anterograde and retrograde degeneration of neural fibers may be a major factor in the reduction of tissue mass in the injured spinal cord (Seif et al., 2007; Yokota et al., 2019). Long-distance retraction of injured axons coincides with the infiltration of monocytes/macrophages, whose phenotypes transition from anti-inflammatory to pro-inflammatory in response to myelin debris (Wang et al., 2015). Direct contact of monocytes/macrophages with the dystrophic endings of insulted axons is considered to be essential to this process (Busch et al., 2009), since the depletion of infiltrating macrophages reduces axonal dieback after SCI (Evans et al., 2014).

## Definition of Regeneration in the Injured Spinal Cord

The term “regeneration” has been used for decades in the field of central nervous system research (Worcester, 1898), but it may be helpful to review what regeneration entails in the recovery process from SCI. The aim in SCI research has been to repair the disrupted neural network to as close to its former status as possible by supporting and enhancing the endogenous potential of sprouting axons and remyelination, which would hopefully lead to the reconnection of descending neural fibers with their original targets such as spinal interneurons and motor neurons in the caudal spinal cord. In cases of severe and complete SCI, in which there is an absence of neuronal substrates for axonal sprouting and spared axons for re-myelination, stem cell transplantation strategies have been employed to replace the cells that have been lost and restore the neural circuitry. Multiple therapeutic approaches focusing on axonal growth, remyelination, cell replacement, and synaptic reorganization have led to functional improvement in SCI animal models, but the majority of studies have not convincingly verified reestablishment of neural circuits. Without confirmation of the changes brought about to the neural pathways governing motor and sensory function, histological regenerative changes to the spinal cord that lead to decreased cavity size, superior axonal growth, or improved myelination, for example, may be peripheral improvements that contribute to functional improvement but may not directly be responsible for it. This black box laying between the histological changes and functional improvement has been the conundrum in the field of SCI research. With technological limits and technical difficulties in convincingly demonstrating changes in neural circuitry, most studies have not attempted its examination and the peer-review process has not required this analysis. However, this may be part of the reason that many treatment strategies demonstrating significant recovery in rodents have failed to reproduce the benefits in human clinical trials. Furthermore, the inherent differences between rodents and humans in regards to spontaneous recovery and anatomical differences concerning the neural pathways increase the complexity of translating the promising effects observed in animal models to the treatment of human SCI patients (Geisler et al., 1991; Casha et al., 2012; Inada et al., 2014).

The recent advances in neuronal tracers have bestowed researchers with the means to investigate the reorganization of neural networks after SCI and to better appreciate the underlying mechanisms that govern the regeneration of injured spinal cords (Kerschensteiner et al., 2005). To reestablish connectivity of neural circuits, neurons need to be reorganized into existing or newly formed neural pathways and oligodendrocytes must myelinate the axons to facilitate electrical transmission (Nashmi and Fehlings, 2001). The pyramidal tract from the brain cortex projecting to the secondary motor neurons in the spinal cord is the main conduct for motor signals, but the propriospinal circuits in the spinal cord have been shown to be crucial for recovery from SCI (Satkunendrarajah et al., 2018), especially in cases of severe and chronic SCI. Considering that treatment strategies for SCI are shifting toward more combinatorial approaches, it is even more important that not only histological changes, but also regeneration of neural connectivity, be examined to explain any improvement of function.

## Components of Spinal Cord Connectivity

**FIGURE 2:**
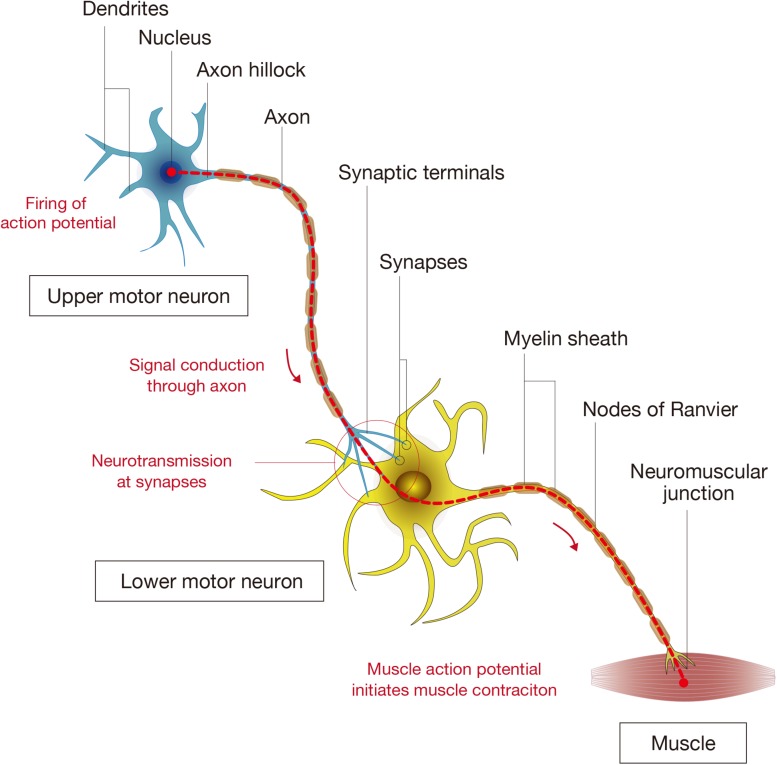
Components of spinal cord connectivity. The diagram shows the simplified components of spinal connectivity composed of an upper motor neuron, a lower motor neuron, and targeted muscle fibers. Neurons have cell bodies, dendrites that receive signals, and axons that transmit signals. At the base of the axon is the axon hillock where the signal transmission is initiated, and the axon divides at its end into several branches, each of which ends in synaptic terminals. The site of interactive communication between a transmitting cell (a presynaptic upper motor neuron) and a receiving one (a postsynaptic lower motor neuron) is called the synapse. The axons of most neurons are covered with a lipid layer knows as the myelin sheath, which insulates axons and speeds up transmission of action potentials through the axon. The axon terminals at a synapse contain tiny vesicles filled with chemicals called neurotransmitters. The lower motor neurons take the impulse to the effector (muscle fibers) and control the coordination of muscle contraction.

The basic unit that allows for the signals governing motion to travel from the brain to the muscles is comprised of a supraspinal neuron that extends a long axon to form a synapse with a motor neuron, whose axon connects to muscle fibers at neuromuscular junctions. The axons are surrounded by myelin sheaths, which allow for the rapid conduction of the electrical signal though the axons (Figure 2; Bellardita and Kiehn, 2015). In the mammalian motor system, the upper motor neurons are located in the brain motor cortex and brainstem while the lower motor neurons are found in the brain stem (cranial motor neurons) and the spinal cord (Le Ray et al., 2011; Roseberry et al., 2016). The actual process of voluntary limb movement and posture control is complex, involving a coordinated synchronous activation of multiple units that are modulated by sensory feedback loops from muscles, tendons, and skin (Bikoff et al., 2016). Furthermore, it is becoming increasingly apparent that propriospinal interneurons that act as bridges between supraspinal neurons and motor neurons play an important role in the plastic reorganization of spinal circuits, contributing an important substrate for recovery from SCI (Courtine et al., 2008). The complicated neuronal networks and the reorganization that takes place in the recovery process from SCI has been partially determined (Shah et al., 2013; Filli et al., 2014), but there is still much that is unknown regarding the interactions between neurons and the surrounding environment that regulate plasticity.

Another important component in neuronal connectivity is the establishment and maintenance of synaptic connections (Williams et al., 2010; Sudhof, 2018). Synapse assembly is a multiple-step process in which immature axons grow and form a physical contact with their target neurons (Kohl et al., 2015). The immature synaptic contacts, both presynaptic and postsynaptic components, undergo a process of stabilization in order to generate and maintain expression levels of neurotransmitters and their receptors to become functional (Kneussel et al., 1999; Easley-Neal et al., 2013). With the growing knowledge that synapses are dynamic structures that are formed and pruned according to multiple factors (Wu et al., 2012; Jacobi et al., 2015; Filipello et al., 2018; Lehrman et al., 2018), they are gaining attention as a subject for intervention. The myelin sheaths formed by oligodendrocytes are another essential component of the neural network and function to enhance transmission of electrical impulses and secrete neurotrophic supports to maintain axonal integrity (Powers et al., 2013; Ishii et al., 2014; Saab and Nave, 2017). Therefore, in order to acquire the neural connectivity necessary for integrated motor and sensory function, upper motor neurons need to be connected to lower motor neurons, possibly mediated by interneurons, through functional synapses and proper myelination.

## Confirming Changes in Spinal Cord Connectivity

The means to evaluate the neural networks of the spinal cord have been available for decades in the form of neural tracers, and it remains the only method to map the fine neural circuits and confirm connectivity. Biotinylated dextran amine (BDA), cholera toxin beta subunit, fluorogold, fast blue, fluorescent microspheres, horseradish peroxidase (HRP), wheat germ agglutinin (WGA), and phaseolus vulgaris-leucoagglutinin (PHA-L) are examples of chemical neural tracers that provide a distinct labeling of neuronal morphology including the neuronal body, dendrites, and axonal terminals (Kuang and Kalil, 1990; Hou et al., 2008; Steencken et al., 2009; Tillakaratne et al., 2010; Sharp et al., 2014; Mondello et al., 2016). With the aid of a stereotaxic apparatus to guide the exact site of the injection, the tracer is injected into selected areas of the brain for anterograde transport and into peripheral organs or spinal segments for retrograde transport, and analyzed in histological sections after a specific period (Chen et al., 2009; Mondello et al., 2016). These tracing methods allow us to visualize the morphology and axonal tract of a specific group of neurons and examine different neurons depending on the amount, sensitivity, and diffusion extent of the tracer. Neurotropic viruses such as herpes simplex virus, lentivirus, and pseudorabies virus were developed to investigate the structural organization of multisynaptic pathways among several neurons (Wickersham et al., 2007; Lo and Anderson, 2011; McGovern et al., 2012; Sheikh et al., 2018). The advantage of this tracing technique is based on the ability of the viruses to transsynaptically enter into connected neuronal cells and self-replicate, providing a means to map multisynaptic neural circuits without signal loss. The disadvantage of viral tracing is the toxicity of the virus to the host neurons, thus limiting studies to approximately 2 weeks. While neural tracers have been available for some time, the technique has been utilized in only a small fraction of researchers examining SCI due to the difficulty in analyzing and interpreting the results. With the growth in knowledge concerning spinal cord neural pathways along with the recent advances in virus engineering to modulate its toxicity, computer technology to create three-dimensional reconstructive images, and software improvements to better trace neuronal tracts, researchers will hopefully be better equipped to analyze the changes that their treatment strategies bring about to spinal cord connectivity. Another recent technique that has been gaining interest is CLARITY (Chung et al., 2013; Tomer et al., 2014), which is a method to chemically transform biological tissue into a transparent hydrogel-tissue hybrid (Yang et al., 2014; Treweek et al., 2015), allowing researchers to perform high-resolution mapping of neuronal networks in combination with viral tracing (Lerner et al., 2015). Neural tracing is a field that is rapidly evolving, and we anticipate that the improved techniques and advances in technology will reveal how our interventions induce plastic reorganizations of neural pathways in SCI, and how the pathways are associated with functional improvements.

While tracing is a well-established method to histologically confirm neural connectivity, electromyograms (EMGs) have been established as a means to non-invasively investigate the functional connectivity of the spinal cord and monitor any longitudinal changes in the same group of animals (Moonen et al., 2016). Motor evoked potentials (MEPs) and somato-sensory evoked potentials (SEPs), which are analyzed in terms of amplitude and latency of the first positive and first negative peaks, provide objective data on spinal cord conductivity with quantitative values and have been shown to predict functional outcomes such as ambulatory capacity and upper limb dexterity. However, it should be noted that reliable MEP and SEP monitoring cannot be obtained in the acute phase of SCI (Feng et al., 2012; Lewis et al., 2017; Dhall et al., 2018). In an evoked EMG, the elicited response includes the H-wave, the M-wave, and the F-wave. The M-wave is the result of direct activation of the motor axons and does not involve the spinal circuits. The later H-wave, or H-reflex, is a compound EMG response in the muscle elicited by synaptic activation of motor neurons through muscle afferents and is regarded as a surrogate for spasticity after SCI. The F-wave is the second voltage change observed after the M-wave, and is the muscle response to the backfire of motor neurons that were stimulated by the antidromic (proximally transmitted) impulses. F waves are often used to measure nerve conduction velocity, and any changes recorded in conduction velocity can reflect the remyelination of neural tracts (Moonen et al., 2016). Longitudinal MEPs, but not SEPs, have been shown to correlate with neurological impairment after SCI (Huang et al., 2018), but their changes may not necessarily be linked with actual phenotypical functional recovery. EMG signals may be useful to verify synaptic connectivity by examining the conduction of electrical impulses through the lesion, but currently cannot be used to examine the regeneration of specific pathways in spinal cord circuits.

Magnetic resonance imaging (MRI) is clinically performed for most SCI patients to diagnose the injury to the cord and vertebral components, plan treatment, and predict prognosis for recovery (Miyanji et al., 2007). With the capability to conduct non-invasive longitudinal studies of an individual subject, MRI is an attractive option to evaluate spinal connectivity (Fehlings et al., 2017). However, since conventional MRI depicts the white matter as uniform tissue and does not have the sensitivity or resolution to depict the complex array of directionally oriented nerve fibers in the spinal cord (Stroman et al., 2012), it becomes necessary to enhance the signals from neural tracts. One such method with a long history is manganese-enhanced MRI, which utilizes manganese ions that are paramagnetic, thus shortening the spin lattice relaxation time constant (T1) of tissue (Martin et al., 2017). Manganese ions are calcium analogs that can enter neurons through voltage-gated calcium channels, be transported along axons by microtubule-dependent axonal transport, and cross synapses to neighboring neurons (Bedenk et al., 2018). Neuronal uptake of manganese ions is activity-dependent, and a study that injected manganese into the cerebrospinal fluid demonstrated that manganese-enhancement was reduced after SCI and that the uptake of manganese ions correlated with functional recovery. Direct injection of manganese ions into the lumbar spinal cord demonstrated enhancement of a wide rostral-caudal area of the thoracic gray matter, demonstrating its possible use to visualize the connectivity of the spinal cord (Stieltjes et al., 2006).

Another more recent innovative use of MRI to visualize neural tracts is diffusion tensor imaging (DTI), which takes advantage of the anisotropic nature of water diffusion in biological tissue to follow the orientation of nerve fibers and trace specific neural pathways, such as the corticospinal tract (CST) (Cheran et al., 2011). DTI has been able to visualize both the intact and injured neural networks of the spinal cord, and the quantitative data from the DTI images was associated with histological findings (Fujiyoshi et al., 2007). When considering the capability of DTI to delineate neural tracts, several limitations of the method need to be understood. The current voxel resolution of 1 to 3 mm in each dimension means that each voxel represents the total anisotropic character of millions of cells, so the images need to be interpreted with the knowledge of this limited resolution (Wheeler-Kingshott et al., 2002). Another factor that affects the results are the effect of free water diffusivity from the cerebral spinal fluid and edema, which contaminates the neuroimaging measurements within a voxel (Maier, 2007; Hoy et al., 2015). However, even with these limitations, the capability to longitudinally visualize changes in spinal cord connectivity make DTI a promising tool, and the advances in imaging scanner technology and diffusion tensor imaging techniques will hopefully increase the value of this method to evaluate the connectivity of the injured spinal cord.

## Key Factors Affecting Regenerative Failure of Spinal Cord Connectivity

### Astrocytic and Fibrotic Scar

After SCI, astrocytes, the most abundant resident cells in the CNS, play a crucial role in SCI pathology through a phenotypic change known as reactive gliosis (Hara et al., 2017). In this process, naive astrocytes undergo a change in phenotype, first as reactive astrocytes and then as scar-forming astrocytes. Immediately after injury, astrocytes proliferate and organize around the edges of the lesion to wall off the damaged area from the surrounding healthy tissue. In the subacute phase (from 1 to 2 weeks after injury), reactive astrocytes migrate to the lesion epicenter and seclude inflammatory cells, leading to tissue repair and functional improvement (Okada et al., 2006; Herrmann et al., 2008; Wanner et al., 2013). Later on, the elongated reactive astrocytes near the lesion perimeter begin to entangle with fibroblast-like pericytes (Goritz et al., 2011; Yokota et al., 2017; Dias et al., 2018), leading to the formation of the astrocytic scar, the main impediment to CNS axonal regeneration (Hara et al., 2017). Although the glial scar was long viewed only as a barrier to CNS regeneration, increasing evidence suggest that the glial scar is necessary to prevent the spread of injury and actually supports CNS repair (Faulkner et al., 2004; Anderson et al., 2016). Indeed, the protective nature of astrocytes were confirmed when complete ablation of astrocytes led to worse outcome after mild to moderate SCI (Bush et al., 1999; Sofroniew, 2009; Burda and Sofroniew, 2014; Anderson et al., 2016). Much has been uncovered concerning the function of reactive astrocytes in SCI, and research is ongoing on how to enhance their beneficial roles while minimizing their deleterious effects.

Although reactive astrocytes have been implicated with most of the inhibitive effects of scarring after SCI, studies have demonstrated the inhibitive effects of a fibrotic scar comprised of a dense extracellular matrix made up of fibronectin, collagen, and fibroblasts. Fibrotic scarring was originally reported to originate from meningeal cells following CNS injury, but recent research has shifted the focus to PDGFRβ-positive pericytes and CD13-positive endothelial cells as an active source of the cellular composition of the fibrotic scar in SCI (Soderblom et al., 2013). Furthermore, a recent study suggested an active role of microvascular endothelial cells in the engulfment of myelin debris through the autophagy-lysosome pathway, which promotes inflammation, angiogenesis, and fibrotic scar formation (Zhou et al., 2019). Although the presence of stromal cells in the scar tissue has been recognized following SCI, their precise origin and role are still not sufficiently elucidated. Further investigation into the origin of the fibrotic scar and the molecular signals leading to its formation may provide potential therapeutic implications for promoting axonal regeneration after SCI.

### Chondroitin Sulfate Proteoglycans (CSPGs)

Chondroitin sulfate proteoglycans (CSPGs), which are growth-inhibitory extracellular matrix glycoproteins that include neurocan, versican, brevican, phosphacan, and NG2 (Jones et al., 2003; Andrews et al., 2012, Anderson et al., 2016), are widely expressed in the CNS and serve as guidance cues during development and modulate synaptic connections in the adult. CSPGs have been shown to repel regenerating axons and also prevent oligodendrocyte maturation and remyelination (Karus et al., 2016). After trauma to the CNS, the inflammatory response upregulates the secretion of CSPGs from astrocytes and non-astrocyte cells, and the accumulated CSPGs become a chemophysical barrier to axonal regrowth, which is regarded as the principle cause for regeneration failure after SCI (Tran et al., 2018b). Degradation of CSPGs by chondroitinase ABC (ChABC) has been shown to be a potential therapeutic strategy to break down the inhibitive barrier and promote endogenous pathological repair, leading to synapse reorganization and functional improvement from SCI (Bradbury et al., 2002). In fact, ChABC in combination with neural stem/progenitor cells (NSPCs) was shown to promote functional recovery even in the chronic phase of SCI (Karimi-Abdolrezaee et al., 2010; Suzuki et al., 2017). CSPG inhibition has been shown to be mediated by two members of the Leukocyte Common Antigen Related (LAR) phosphatase subfamily, protein tyrosine phosphatase σ (PTPσ) and LAR, and PTPσ receptors have been shown to mediate the regulation of oligodendrocyte differentiation and apoptosis by CSPGs in the injured spinal cord (Fisher et al., 2011; Dyck et al., 2018). Recent studies have demonstrated that administration of a blocking peptide for the CSPG receptor PTPσ restored neuronal innervation of the pyramidal tract projecting to secondary motor neurons and led to functional recovery (Lang et al., 2015; Tran et al., 2018a). The evidence showing that ChABC is beneficial to functional recovery from SCI is growing, but there remain many obstacles that need to be overcome before ChABC treatment can be clinically applied to SCI. The low thermal stability and the short half-life of ChABC make it necessary to repeatedly or continually administer the drug through invasive channels (Lee et al., 2010), and its bacterial origin raises concerns about its safety and immunogenicity (Prabhakar et al., 2009). Animal studies performed so far have not shown adverse effects of ChABC treatment, but the long-term effects of ChABC administration need to be investigated before clinical application can be considered. In order to sidestep some of the disadvantages of ChABC, recent studies are looking into gene therapies to engineer ChABC expression in the injured spinal cord (James et al., 2015; Burnside et al., 2018). Although gene therapy is still an evolving process and methods to control transgene expression require further refinement, progress in this field may 1 day make this option favorable to administration of ChABC.

### Inflammatory Reaction

After SCI, the intensive local inflammatory response leads to the activation of resident microglia and facilitates the infiltration of macrophages into the lesion (Shechter et al., 2009). The CNS has been traditionally considered an immune-privileged site and the inflammatory storm that occurs in the early phases of SCI was considered detrimental to spinal cord function, but the contribution of immune cells to the healing process has also been revealed. One of the main players in the inflammation process is macrophages, and they have been described as having pro- (M1) or anti-inflammatory (M2) functions (Donnelly et al., 2011; Shechter et al., 2013). This grouping of macrophages into M1 and M2 groups may be an oversimplification with macrophages actually being somewhere on this spectrum of polarization, but this bimodal characteristic of infiltrating macrophages has improved our understanding of their function in the injured spinal cord (Kroner et al., 2014; Wang et al., 2015). With systemic and localized inflammatory reactions persisting from the acute to chronic phase of SCI (Ulndreaj et al., 2017; Badner et al., 2018; Hong et al., 2018), interventions that modify inflammation hold promise as a means to reduce secondary damage after SCI (Nguyen et al., 2012; Badner et al., 2016), and modulating the phenotypes of the infiltrating macrophages may be a therapeutic strategy to promote functional recovery after SCI.

### Syringomyelia

The overwhelming cell death and tissue degeneration from the acute to chronic phases after SCI promotes the loss of parenchymal tissue at the lesion epicenter and leads to the formation of cystic cavities referred to as syringomyelia (Seki and Fehlings, 2008). Although the pathological mechanisms underlying syringomyelia progression in CNS trauma is not completely understood, the process of posttraumatic cavitation is found in both humans and mammals. The cystic cavities that form following SCI contain extracellular fluid, bands of connective tissue, and infiltrated monocytes/macrophages (Austin et al., 2012a), and the increasing cerebral spinal fluid (CSF) pressure within the cavity is detrimental for regeneration and acts to enlarge its size. The cavity is a formidable obstacle that regenerating axons need to overcome in order to reconnect with their severed networks, prompting researchers to consider strategies that would modify this gap in the spinal cord into a growth-enhancing medium that would nurture regenerating axons and encourage reinstatement of spinal cord connectivity.

## Therapeutic Approaches to Overcome Obstacles in the Lesion Core and Promote Regeneration

### Cell-Based Therapies

**FIGURE 3:**
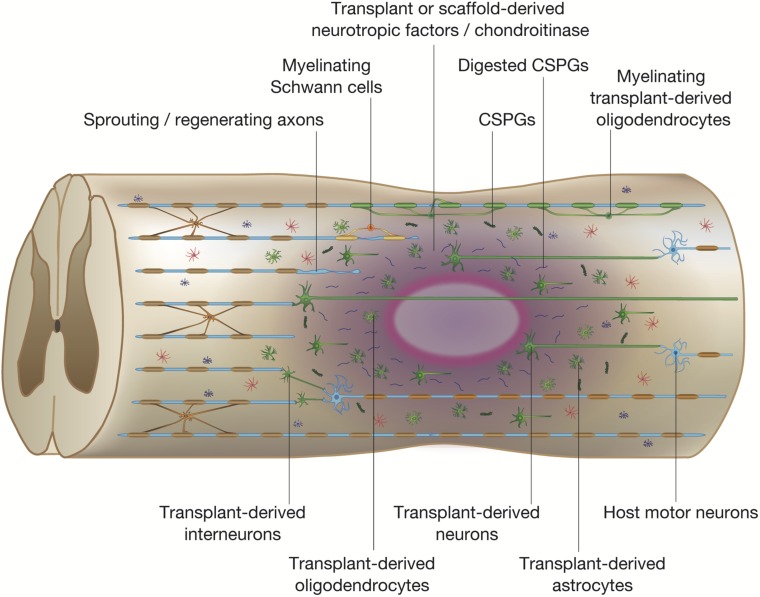
Potential mechanisms of spinal cord repair by stem cell transplantation. The diagram shows potential mechanisms of regeneration brought about by stem cell transplantation. The transplanted stem cells differentiate into neural cells of the three lineages: neurons, astrocytes, and oligodendrocytes (shown in green). The transplanted stem cells and differentiated cells secrete neurotrophic factors that reduce inflammation, degrade CSPGs, and promote endogenous tissue repair. Differentiated oligodendrocytes remyelinate denuded axons. The grafted neurons form synapses with propriospinal neurons and lumbar motor neurons, which reorganize the neuronal circuits by forming *de novo* synaptic connectivity between host and grafted neurons. The regenerated neuronal circuits bridge the lesion by creating a detour route that passes through areas more favorable to regenerating axons. Transplant-derived interneurons indirectly connect the host injured neural tracts through the propriospinal circuits, whereas transplant-derived neurons participate in the regeneration of the injured corticospinal tract (CST) and directly activate muscle contraction.

Considering the extensive loss of neural cells after SCI, transplantation of various types of cells into the injured spinal cord to repopulate cells that are not replenished by the endogenous regenerative process is an obvious strategy to treat SCI. We now know that engrafted cells work not only by repopulating cells, but by modulating the transplantation site into a more hospitable environment that prevents demyelination and apoptosis of neural cells (Figure 3). Of the numerous candidates for transplantation, NSPCs, which are multipotent CNS cells capable of differentiating into neurons, astrocytes, and oligodendrocytes, have been the most attractive and well-studied cell source for the treatment of SCI (Wilcox et al., 2014). While we recognize that neural stem cells, neural progenitor cells, and neural precursor cells are, strictly speaking, different cell populations, we also believe that most cell transplants are a mix of these cells. Therefore, in the interest of clarity, we have elected to unify the designation of these cells as NSPCs. Following transplantation, engrafted NSPCs differentiate into neural cells that replace damaged cells and provide local neurotrophic factors that support neuroprotection, immunomodulation, axonal sprouting, axonal regeneration, and remyelination. Embryonic stem cells (ESCs) were once considered to be a promising candidate for transplantation due to their unlimited developmental potential, but safety concerns associated with their tumorigenicity have greatly deflated the enthusiasm surrounding ESCs and research has shifted more to ESC-derived NSPCs, which have demonstrated therapeutic potential as a treatment for SCI (Salewski et al., 2015b).

Recent advances in stem cell engineering have led to the development of directly reprogrammed NSPCs from human fibroblasts, blood cells, and mesenchymal cells, and they have demonstrated their potential to promote axonal remyelination and tissue sparing in mammal SCI models (Nagoshi et al., 2018). With the possibility for autologous transplantations that would eliminate the risk of an immune response against the transplanted cells, directly reprogrammed NSPCs are an attractive cell source for transplantation treatments. Induced pluripotent stem (iPS) cells and iPS cell-derived neural stem cells (iPS-NSCs) are currently at the forefront of stem cell transplantation strategies, and recent studies show that iPS-NSCs transplanted into the injured spinal cord contribute to remyelination of axons, secretion of regenerative neurotrophic factors, and synaptic reorganization (Salewski et al., 2015a). However, one of the issues that needs to be addressed is tumorigenicity, which is a potential problem with all stem cell transplantations, but it has been most closely studied in iPS cell lines due to its imminent clinical application. The tumorigenicity of iPS cells was reviewed in a recent report (Deng et al., 2018), and methods to eliminate iPS cell-derived tumors are being refined (Kojima et al., 2019).

## How Engrafted Stem Cells Contribute to Spinal Cord Connectivity

### Remyelination

Remyelination in the CNS is a dynamic process that begins with the proliferation of OPCs and their differentiation into oligodendrocytes, which then ensheath axons. A portion of the NSPCs transplanted in the acute or subacute phases of SCI differentiate into oligodendrocytes, increase the number of myelinated axons around the lesion, and lead to functional improvements (Karimi-Abdolrezaee et al., 2006; Eftekharpour et al., 2007). A previous report showed that NSPCs harvested from shiverer rodents, which have severe myelin deficiency throughout the CNS, were less effective than those harvested from wild mice-derived NSPCs when transplanted into the injured spinal cord of wild-type rodents (Yasuda et al., 2011; Hawryluk et al., 2014). These studies reveal that NSPC-derived myelin is essential to the remyelination process after SCI, and demonstrate the important role that remyelination plays in the functional recovery brought about by stem cell transplantation strategies to treat SCI.

With demyelination playing a large role in functional impairment after SCI (Nashmi and Fehlings, 2001; Sinha et al., 2006; Ouyang et al., 2010; Papastefanaki and Matsas, 2015), replacing lost oligodendrocytes through oligodendrocyte precursor cell (OPC) transplantation is another strategy that is being studied (Keirstead et al., 2005). OPCs are predominantly quiescent in the healthy CNS, but in response to injury they proliferate and differentiate into mature oligodendrocytes, which contribute to remyelination (Assinck et al., 2017). Transplanted OPCs not only complement the insufficient remyelination process of endogenous OPCs, but also secrete neurotrophic factors that ameliorate inflammation and promote axonal regeneration (Zhang et al., 2006; Sharp et al., 2010). Schwann cells (SCs), which myelinate peripheral nerve fibers, have also been shown to migrate into the injured spinal cord and support remyelination after SCI (Pearse et al., 2004; Hill et al., 2006). Being more accessible for harvest and easier to culture compared to OPCs, SCs are another attractive cell source to promote remyelination (Anderson et al., 2017). Transplanted SCs have been shown to remyelinate axons and improve neural conduction similar to OPCs, and are reported to produce growth factors, extracellular components, and adhesion molecules that promote functional recovery after SCI (Golden et al., 2007; Papastefanaki et al., 2007; Cao et al., 2010; Lavdas et al., 2010; Deng et al., 2013).

### Axonal Sprouting

The dysfunction after SCI is caused mainly by the disruption of functional connections around the lesion site. The cavity that forms in the injured spinal cord lacks the substrate necessary for axonal sprouting and is an impediment for endogenous tissue repair, but the lesion cavity as well as the tissue around the lesion are reasonable engraftment sites for transplanted cells. Stem cells transplanted into the cavity and/or surrounding tissue engraft and secrete neurotropic factors that promote the growth of axons, both endogenous and graft-derived, across the lesion to form synapses and restore spinal cord connectivity (Lu et al., 2014). Even after decades of research into stem cell transplantation therapies, however, it is still a challenge to induce robust axonal growth that spans the lesion cavity and forms functional connections with the remaining neural network, mainly due to the low regenerative capacity of the injured spinal cord and its refractory environment. Therefore, it is becoming increasingly more frequent to combine stem cell transplantation with other strategies that would enhance the effect of the transplanted cells (Ruff et al., 2012). Biomaterial scaffolds, which provide a growth-permissive substrate for axons to grow, are a logical option to accompany stem cells and will be described later. The scaffolds are often bioengineered to secrete growth-enhancing neurotropic factors, and stem cells are often genetically manipulated to secrete factors that break down growth-inhibitive barriers or promote axonal growth.

### Promotion of Neural Pathway Plasticity

One of the mechanisms through which functional improvements occur in subjects with SCI is through neural plasticity, or the ability of the CNS to reorganize its circuits over time (Adler et al., 2017; Wang et al., 2018a). These adaptive changes may occur at any level within the spared neuronal circuitry: the motor cortex, brainstem, or spinal cord level, both above and below the lesion (Bareyre et al., 2004; Courtine et al., 2008). The neurons that differentiate from engrafted NSPCs extend axons and form new synapses with host neurons; the established connections are generally not exact reconnections of the lost neural circuits, but rather *de novo* circuits (Bonner et al., 2011). This reorganization is a very dynamic and variable process, and its degree is believed to depend on the age of the subject and the rehabilitative therapy. Utilizing retrograde neuronal tracing and drug-induced ablation of host neurons, it was demonstrated that the reorganized propriospinal circuits generated through synaptic formation between graft-derived neurons and host-derived neurons directly contributed to functional recovery after NSPC transplantation (Yokota et al., 2015). However, the neural plasticity brought about by NSPC transplantation and its specific role in reestablishing spinal cord connectivity remain ambiguous due to the lack of information regarding the spatial and temporal integration of transplanted stem cells into the host neural circuitry.

While the plasticity of neural circuits in the injured spinal cord has long been proposed to be one of the mechanisms leading to functional recovery from SCI, many studies have only presented fragmentary circumstantial evidence of plasticity. Indeed, the burden of proving plasticity is high because, ideally, one would need to present tracing results to show the pathways before and after SCI and demonstrate functional transference of the microcircuitry from one pathway to another through functional and/or electrophysiological studies. A recent study from our group convincingly demonstrated the plasticity of cervical neural circuits involved in the control of respiration in SCI. In both traumatic (C2 hemisection) and non-traumatic (cervical myelopathy) SCI models, respiratory control shifted from phrenic motor neurons that normally control diaphragm motion to mid-cervical excitatory interneurons, which are normally not essential for the maintenance of breathing in healthy animals. The selective silencing of these excitatory interneurons led to severe disruption of the animals’ ability to maintain breathing, indicating their crucial role in respiratory plasticity after SCI (Satkunendrarajah et al., 2018). With increasing attention being paid to the vital role that plasticity plays in maintaining or reestablishing connectivity of the injured spinal cord, the future use of precise neuronal tracing, sophisticated image reconstruction technology, and genetic techniques that manipulate functionality will hopefully elucidate the contribution of plasticity to recovery from SCI.

### Stimulation of Endogenous Stem Cells

Ependymal cells, which are the ciliated cells lining the central canal of the spinal cord, are responsible for the propulsion of cerebrospinal fluid and function as a barrier to the spinal cord parenchyma. The normally quiescent ependymal cells self-renew in response to SCI and differentiate into oligodendrocytes and astrocytes (Ke et al., 2006; Barnabe-Heider et al., 2010). The significance of the ependymal cell-derived cell population was confirmed when inhibition of ependymal cell proliferation after SCI severely compromised glial scar formation and led to increased neuron loss (Sabelstrom et al., 2013). Furthermore, harvested and cultured ependymal cells are capable of differentiating into astrocytes, oligodendrocyte, and neurons. Altogether, the characteristics of ependymal cells demonstrate that they are the endogenous stem cells in the adult spinal cord and therefore constitute an attractive cell population to target in the treatment of SCI (Johansson et al., 1999; Yamamoto et al., 2001; Meletis et al., 2008). Indeed, infusion of the growth factors EGF and FGF2 into the central canal was shown to increase the proliferation of ependymal cells and improve functional recovery after SCI, demonstrating the potential of ependymal cell manipulation as an alternative to exogenous stem cell transplantation (Kojima and Tator, 2002).

Additionally, there is experimental data showing that exogenous stem cell transplantation induces proliferation of the endogenous stem cell pool in ependymal cells. Neural stem cells transplanted into the lumbar ventral horn migrated to the central canal and have been shown to stimulate proliferation of ependymal cells and their differentiation into neural precursors and neurons (Xu et al., 2012). The results of this study suggest that transplanted exogenous neural stem cells may induce neurogenesis in the spinal cord ependymal niche and also promote survival of the newly generated host neurons, which is similar to the neurogenesis induced in the brain subventricular zone by NSPC and mesenchymal stem cell grafts (Bao et al., 2011; Jin et al., 2011). If stem cell transplants could be engineered to further stimulate the proliferation of ependymal cells, the synergistic effect between the transplanted exogenous stem cells and endogenous stem cells may bring about greater recovery compared to either stem cell population alone. However, research into the endogenous stem cells of the spinal cord is insufficient to reliably understand and harness this stem cell population, and we await further studies to deepen our knowledge on the potential of ependymal cells.

## Biomaterial Scaffolds

### Overview of Biomaterials

With the large cavity forming after SCI being an obstacle for regenerating axons, there have been many attempts to implant constructs into the cavity to provide axons with a substrate on which to grow and to restore tissue continuity across the trauma zone. These attempts started as oriented structures to act as bridges for growing axons, but have since evolved to secrete factors that enhance tissue growth and vascularization, deliver drugs, and act as a vehicle to deliver cells into the lesion (Elliott Donaghue et al., 2014). Scaffolds can be designed as devices for controlled release of therapeutic drugs, which would replace the need for multiple and high-dose drug administration (Pakulska et al., 2016b). Many different types of scaffolds have been developed for the treatment of SCI (Liu et al., 2013), but based on composition they can be classified as natural polymers, synthetic biodegradable polymers, or synthetic non-degradable polymers. Being derived and purified from biological sources, natural polymers are biodegradable, have natural biding sites for cells, and generally elicit lower inflammatory reaction and immune response (Tam et al., 2014). Being the product of chemical bioengineering, synthetic biomaterials allow for greater product consistency and tunable properties compared to natural ones (Pakulska et al., 2015, 2016a). Many biomaterial substrates have been studied as candidate scaffolds for the treatment of SCI: collagen, laminin, fibrin matrices, fibronectin, hyaluronan-methylcellulose, chitosan, agarose, alginate, methylcellulose, poly(2-hydroxyethyl methacrylate) or pHEMA, poly(N-(2-hydroxypropyl) methacrylamide) or pHPMA, and poly(lactic-co-glycolic) acid or PLGA. Each substrate has its advantages and disadvantages, and there is currently no consensus on the substrate of choice (Haggerty and Oudega, 2013). The ideal scaffold would have a simple design that allows for smooth manufacturing, have good biocompatibility with low immunogenicity, be biodegradable, have mechanical properties ideal for cell adhesion and axonal regeneration, and would be easy to transplant into the injured spinal cord. Focusing on the ease of transplantation into the SCI cavity, form-filling injectable hydrogel polymers have been receiving attention, and studies have shown that hydrogels decrease cavitation, improve engraftment of transplanted cells, and provide sustained delivery of neurotrophic agents (Austin et al., 2012b). The treatment strategies for SCI have been shifting toward a combinatorial approach, and with the many beneficial characteristics provided by biomaterial scaffolds (Pawar et al., 2015; Chedly et al., 2017; Ropper et al., 2017; Santhosh et al., 2017; Ghosh et al., 2018; Oudega et al., 2018), it is not surprising that many studies have incorporated scaffolds into their treatment paradigms.

### How Biomaterial Scaffolds Contribute to Spinal Cord Connectivity

The microenvironment of the SCI lesion is inhibitive to regeneration, and biomaterial scaffolds are implanted in the hopes of improving the lesion into a more growth-supportive environment that would support endogenous neurogenesis, axonal sprouting, and neural plasticity. Scaffolds provide contact-mediated guidance for aligned axon growth across the lesion site and act as a vehicle to deliver drugs and biomolecules that favorably modify the environment as well as stem cells that repopulate the lost neural cells.

A recent study reported on the positive effects of transplanting chitosan, a porous hydrogel scaffold, loaded with neutrotrophin-3 (NT-3) into the SCI lesion of adult rats or rhesus monkeys. The chitosan scaffold effectively prevented infiltration of inflammatory cells, attracted endogenous neural stem cells to proliferate, migrate, and differentiate into neurons, and facilitated the reorganization of neural relay networks to transmit ascending and descending neural signals (Yang et al., 2015). Diffusion tensor imaging, functional MRI, electrophysiology, and kinematics-based quantitative walking behavioral analyses were employed to confirm the robust neural regeneration that led to significant motor and sensory functional recovery (Rao et al., 2018). Diffusion tensor imaging, functional MRI, electrophysiology, and kinematics-based quantitative walking behavioral analyses were employed to confirm the robust neural regeneration that led to significant motor and sensory functional recovery. Anterograde neuronal tracing revealed that axons of the corticospinal tract (CST) regenerated through the grafted scaffold into the caudal part of the spinal cord, and electrophysiology confirmed restoration of MEP signals by the regenerated neural tissue, demonstrating partial restoration of spinal connectivity.

Another recent study performed by Sofroniew’s group strategically used injected hydrogels, termed biomaterial depots, to achieve sustained delivery of growth factors. These biomaterial depots were prepared using diblock copolypeptide hydrogels that are biocompatible with the CNS, biodegrade over several weeks, and provide delivery of bioactive growth factors for at least 2 weeks (Yang et al., 2009; Song et al., 2012). Adeno-associated viral vectors (AAV) were injected 2 weeks before injury to reactivate intrinsic propriospinal neuronal growth capacity through phosphatase and tensin homologue (PTEN) knockdown or by expressing osteopontin, insulin-like growth factor 1 (IGF1) and ciliary-derived neurotrophic factor (CNTF). After inducing a severe crush SCI, biomaterial depots delivering fibroblast growth factor 2 (FGF2) and epidermal growth factor (EGF), in combination with and without glial-derived neurotrophic factor (GDNF) or an integrin-function-blocking antibody, were injected into the spinal cord. The authors demonstrated that by sequentially reinstating several developmentally essential mechanisms that facilitate axon growth, it is possible to induce robust growth of propriospinal axons across anatomically complete SCI lesions in adult rodents (Anderson et al., 2018). BDA tract-tracing of propriospinal neurons demonstrated that axons regenerated across the lesion and formed synapses that conveyed a significant return of electrophysiological conduction capacity across the lesion. Although the intervention did not elicit supraspinal serotonergic axonal regeneration or result in observable functional recovery, possibly due to the severity of the injury and the lack of rehabilitation that promotes neural pathway plasticity, this study demonstrates how biomaterials can be utilized to restore spinal connectivity.

## Combinatorial Therapies Including Neural Stem/Progenitor Cell Transplantation and Biomaterial Scaffolds

While the transplantation of stem cells and scaffolds have each demonstrated beneficial effects as sole treatments, there are numerous studies reporting the synergistic enhancements elicited by combining these two methods (Li et al., 2013). Some selected studies using combinatorial treatment strategies are outlined in Table 1. Our group has been studying the benefits of combining NSPCs and K2(QL)6K2 (QL6), which is an aqueous self-assembling peptide (SAP) that aggregates into a stable nanofiber gel due to multiple non-covalent interactions. When injected by itself into the injured spinal cord, QL6 reduced neural cell apoptosis, inflammation, and astrogliosis and brought about electrophysiological and behavioral improvements (Liu et al., 2013). The combination of SAP injection and NSPC transplantation improved NSPC engraftment, reduced astrogliosis and CSPG deposition, increased synaptic connectivity, and improved behavioral outcomes compared to sole treatments (Zweckberger et al., 2016).

**TABLE 1:** Selected studies using a combinatorial therapy comprised of neural stem cell transplantation with a biomaterial containing neuroprotective agents.

**Author**	**Year**	**Cell source**	**Biomaterial**	**Neurotrophic agents**	**SCImodel, species**	**Results**
Koffler	2019	Rat spinal cord-derived NSPCs	3D biomimetic hydrogel scaffolds including GelMa, PEGDA, and LAP	Growth factor cocktail (BDNF, VEGF, bFGF, calpain inhibitor)	T3 complete transection, Fischer rats	The injured host axons regenerated into 3D biomimetic scaffolds and synapsed onto NSPCs implanted into the device, and implanted NSPCs extended axons out of the scaffold and into the host spinal cord below the injury to restore synaptic transmission and significantly improve functional outcomes.
Rosenzweig	2018	Human spinal cord-derived NSPCs	Fibrin matrix	Growth factor cocktail (BDNF, NT-3, GDNF, EGF, bFGF, aFGF, HGF, IGF-1, VEGF, PDGF-AA, calpain inhibitor)	C7 right lateral hemisection, rhesus macaques (Macaca mulatta)	Grafted axons extended through host white matter and synapsed in distal gray matter. Grafts gradually matured over 9 months and improved forelimb function beginning several months after grafting.
Nori	2018	Human directly reprogrammed drOPCs	Thiolated methylcellulose modified with SH3 domain binding peptides	Recombinant ChABC-SH3 fusion protein	T7 clip injury, RNU (athymic nude) rats	This combinatorial therapy increased long-term survival of drOPCs around lesion epicenter and facilitated greater oligodendrocyte differentiation, which led to remyelination of the spared axons by engrafted drOPCs and enhanced synaptic connectivity with anterior horn cells, leading to neurobehavioral recovery.
Kadoya	2016	Rat spinal cord-derived NSPCs	Fibrin matrix	Growth factor cocktail (BDNF, NT-3, PDGF-AA, IGF-1, EGF, bFDF, aFGF, GDNF, HGF, calpain inhibitor)	T3 complete transection and C4 (CST) lesion, Fischer rats	Grafted cells showed robust corticospinal axon regeneration that formed functional synapses and led to improvement in skilled forelimb function.
Führmann	2016	Human iPSC-derived OPCs	Hydrogel blend of hyaluronan and methylcellulose (HAMC)	RGD (arginine-glycine-aspartic acid) peptide, PDGF-A	T2 clip injury, Sprague Dawley rats	HAMC hydrogel, modified with a RGD peptide and PDGF-A, promoted early survival and integration of grafted cells. Teratoma formation was attenuated when cells were transplanted in the hydrogel, where most cells differentiated to a glial phenotype.
Mothe	2013	Rat brain-derived NSPCs	Hydrogel blend of hyaluronan and methylcellulose (HAMC)	Recombinant PDGF-A (rPDGF-A)	T2 clip injury, Wistar rats	SCI rats transplanted with NSPCs in HAMC-rPDGF-A showed improved behavioral recovery compared to rats transplanted with NSPCs in media. NSPC/HAMC-rPDGF-A group had significantly reduced cavitation, improved graft survival, increased oligodendrocytic differentiation, and increased sparing of perilesional host oligodendrocytes and neurons.
Li	2013	Rat brain-derived NSPCs	Collagen scaffolds	EGFR neutralizing antibody	T13-L2 lateral hemisection, Sprague Dawley rats	The scaffold loaded with the EGFR antibody neutralized the negative effects of myelin proteins and directed the differentiation of transplanted NSPCs to a neuronal lineage, which promoted functional recovery after SCI.

*NSPCs, neural stem/progenitor cells; GelMa, gelatin methacrylate; PEGDA: poly(ethylene glycol) diacrylate; LAP, lithium phenyl-2,4,6-trimethylbenzoylphosphinate; BDNF, brain derived neurotrophic factor; NT-3, neurotrophin-3; GDNF: glial cell-derived neurotrophic factor; EGF, epidermal growth factor; bFGF, basic fibroblast growth factor; aFGF, acidic fibroblast growth factor; HGF, hepatocyte growth factor; IGF-1, insulin-like growth factor-1; PDGF, platelet-derived growth factor; VEGF, vascular endothelial growth factor; drOPCs, directly reprogrammed oligodendrogenic progenitor cells; ChABC, chondrotinase ABC; SH3, Src homology 3; CST, corticospinal tract; SCI, spinal cord injury; iPSCs, induced pluripotent stem cells; HAMC, hyaluronan methylcellulose; RGD, arginine-glycine-aspartic acid; EGFR, epidermal growth factor receptor.*

Current treatment strategies now often combine scaffolds and stem cells with enhancements bioengineered into the scaffolds, cells, or both. In a study that explored the modification of a scaffold with platelet-derived growth factor-A (PDGF-A) to induce oligodendrocyte differentiation, NSPCs cultured in a hydrogel blend of hyaluronan and methylcellulose (HAMC) modified with PDGF-A had improved survival and a higher percentage of cells differentiating into oligodendrocytes. SCI rats transplanted with NSPCs in HAMC-PDGF-A showed reduced cavitation, improved graft survival with increased oligodendrocytes differentiation, and improved behavioral recovery compared to rats transplanted with NSPCs in media (Mothe et al., 2013). The authors further modified the HAMC-PDGF-A scaffold with arginine-glycine-aspartic acid (RGD) peptide to improve the survival and engraftment of human iPS cell-derived OPCs. Compared to iPS cell-derived OPCs transplanted with media, iPS cell-derived OPCs transplanted in HAMC-RGD/PDGF-A had higher rates of survival and engraftment. Interestingly, while all animals that received cells in media formed teratomas, cells injected in HAMC-RGD/ PDGF-A only formed teratomas in half of the animals, demonstrating that the modified hydrogel promoted cell differentiation and attenuated tumor formation (Fuhrmann et al., 2016). These studies demonstrate the large effects that scaffold modifications can have on the survivability of transplanted cells and its characteristics after engraftment into the injured spinal cord.

Some of the most dramatic synergistic effects of scaffolds, stem cells, and growth factors have been reported by Tuszynski’s group. In a report examining the effects of transplanting spinal cord-derived NSPCs into a rat thoracic cord transection model, NSPCs transplanted alone engrafted only on the lesion margin. When the same cells were transplanted in fibrin matrix containing a cocktail of growth factors (brain-derived neurotrophic factor, neurotrophin-3, glial-cell-line-derived neurotrophic factor, epidermal growth factor, basic fibroblast growth factor, acidic fibroblast growth factor, hepatocyte growth factor, insulin-like growth factor, platelet-derived growth factor, vascular endothelial growth factor, and a calpain inhibitor), the transplanted NSPCs filled the lesion gap and demonstrated robust axonal growth caudally into the host spinal cord. The axons from the engrafted NSPCs formed synapses that led to improved electrophysiological and functional improvements (Lu et al., 2012). A following study that examined the regeneration of the corticospinal tract (CST) by transplanting NSPCs and the growth cocktail-enhanced fibrin matrix into a similar rat transection model demonstrated robust CST axon regeneration across the lesion that formed functional synapses and led to improved forelimb function. However, this regeneration was observed only when the grafts were caudalized NSPCs or primary spinal cord–derived NSPCs, demonstrating that the characteristics of the graft were a vital ingredient for CST regeneration (Kadoya et al., 2016). With the aim of generating translational data, the group then studied the effects of transplanting human spinal cord–derived NSPCs and the growth cocktail-enhanced fibrin matrix into sites of cervical SCI in rhesus monkeys. Although modifications of the grafting technique and immunosuppression were required, the human NSPCs grafted into the monkey spinal cord extended long axons through the host white matter that formed synapses in the caudal lumbar gray matter, and led to improved forelimb function (Rosenzweig et al., 2018). In the group’s most recent report, the authors created complex 3D biomimetic CNS scaffolds composed of polyethylene glycol-gelatin-methacrylate (PEG-GelMa) based on images of the rat spinal cord (Koffler et al., 2019). Spinal cord-derived NSPCs suspended in a fibrin matrix containing brain-derived neurotrophic factor, basic fibroblast growth factor, vascular endothelial growth factor, and a calpain inhibitor were loaded into the scaffolds and inserted into a rat thoracic cord transection lesion. The transplanted NSPCs survived and filled the scaffold channels at 1 month, and the scaffolds maintained their 3D architecture 6 months after implantation. Host axons regenerated into the scaffolds and formed synapses with NSPCs in the scaffold, while engrafted NSPCs extended axons into the host spinal cord and restored synaptic transmission, leading to electrophysiological and functional improvements. These studies show that with the appropriate combination of optimally engineered stem cells, scaffolds, and growth factors, the hostile environment of the SCI lesion can be improved and neural cells of the spinal cord can be coaxed into a state of regeneration.

**FIGURE 4:**
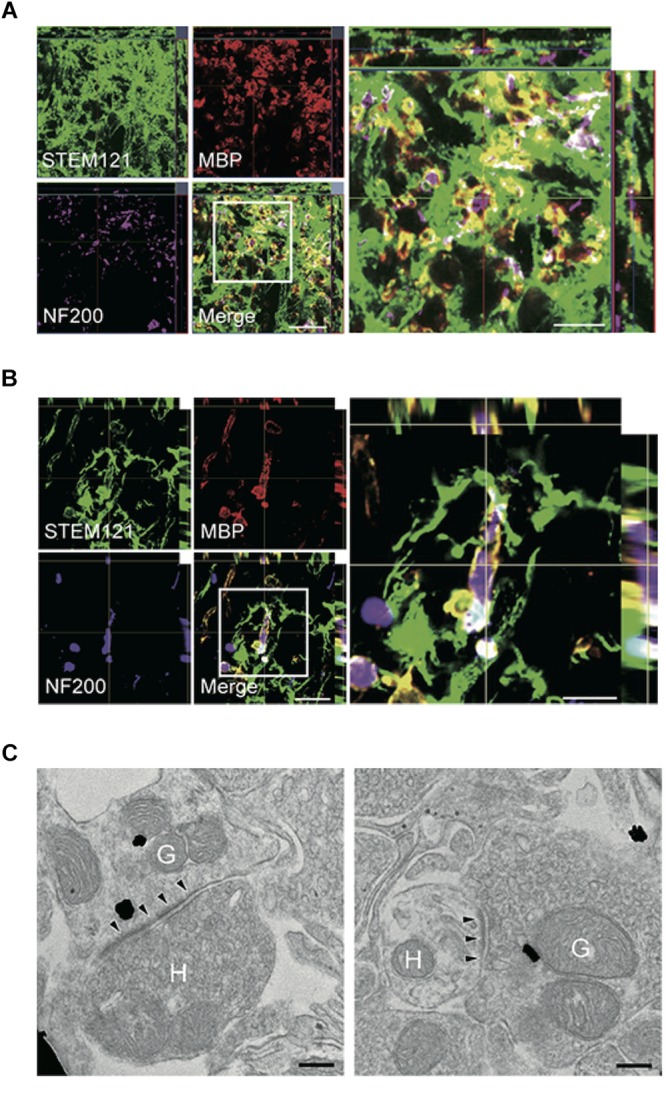
Combinatorial treatment of stem cells and biomaterials containing ChABC elicits remyelination and synaptic reorganization. **(A,B)** Representative images of axial **(A)** and sagittal **(B)** sections stained for STEM121 (a specific marker for human cytoplasmic protein; green), MBP (red), and NF200 (magenta). Many STEM121-positive/MBP-positive graft-derived myelin sheathes were observed around NF200-positive host axons. **(C)** Immunoelectron microscopy images show synapses formed between host and graft-derived neurons after the combinatorial treatment. Presynaptic and postsynaptic structures indicate transmission from host neurons to graft-derived neuron (left image), and from graft-derived neurons to host neurons (right image). Annotated (H) indicates host neurons, and (G) indicates graft-derived neurons. Arrowheads indicate postsynaptic density. Figure altered with permission from Nori et al. (2018). Human oligodendrogenic neural progenitor cells delivered with chondroitinase ABC facilitate functional repair of chronic spinal cord injury (2018).

Especially for the treatment of chronic SCI, a combinatorial approach is believed to be the only possible avenue to reactivate the regenerative processes and gain functional improvements. Previous reports showed that a combinatorial treatment strategy using stem cells and ChABC promoted functional recovery in the chronic phase of SCI (Karimi-Abdolrezaee et al., 2010; Suzuki et al., 2017; Nori et al., 2018), demonstrating that ChABC treatment can modify the chronically injured spinal cord into a microenvironment conducive to regenerative cell-based therapy. After ChABC was administered by intrathecal injection of a methylcellulose hydrogel containing ChABC, human-derived directly reprogrammed oligodendrocyte progenitor cells (drOPCs) were transplanted into the injured spinal cord of rats. ChABC was administered with the intent to degrade CSPGs and also to maintain the oligodendrocytes profile of the drOPCs (Karimi-Abdolrezaee et al., 2012). The transplanted drOPCs enhanced synapse formation, promoted remyelination of host axons, and improved functional recovery (Nori et al., 2018). They found that graft-derived cells formed a MBP-positive myelin sheath and enwrapped host spared axons in the chronically injured spinal cord (Figures 4A,B). Using immunoelectron microscopy, they also revealed that immunogold-labeled differentiated graft-derived neurons formed synaptic connectivity with host neurons (Figure 4C). This study demonstrated that with an appropriate combinatorial therapy including ChABC and stem cell transplantation, regeneration in the chronically injured spinal cord is also possible.

## Conclusion and Future Perspectives

As we have outlined in this review, significant progress has been made in the recent decades to elucidate the pathophysiology of SCI and to uncover the mechanisms that make the injured spinal cord refractory to regeneration. By modulating inflammation, repopulating lost neural cells through transplantation, improving the local environment by implanting biomaterial scaffolds with growth factors, and implementing strategies to break down the inhibitory barriers, impressive recovery has been demonstrated in animal models of SCI. Yet it is important to keep in mind that all interventions must bring about an improvement in neural connectivity for any meaningful improvement to occur. The ongoing progress seen in neural tracing procedures, electrophysiological techniques, as well as imaging hardware and software has improved our understanding of the plasticity of neural circuits following SCI and the importance of propriospinal circuits in the restoration of neural connectivity, but at the same time, the increasing knowledge emphasizes our lack of control on the processes that govern the rewiring of pathways. Since aberrant rewiring has been implicated in mechanical allodynia, we must learn how to establish control of plasticity and not just blindly promote it. As more SCI studies begin to examine changes in spinal cord connectivity and the mechanisms underlying the rewiring of circuits and synapses, therapies that harness and enhance plasticity to promote the recovery from SCI will hopefully be developed in the near future.

## Author Contributions

HK and KY reviewed the literature, wrote and edited the manuscript, and finalized and approved the manuscript. MF conceived the frame and reviewed, edited, finalized, and approved the manuscript.

## Conflict of Interest Statement

The authors declare that the research was conducted in the absence of any commercial or financial relationships that could be construed as a potential conflict of interest.
